# Weight-for-Height, Body Fat, and Development in Children in the East Asia and Pacific Region

**DOI:** 10.1001/jamanetworkopen.2021.42458

**Published:** 2022-01-06

**Authors:** Fanny Petermann-Rocha, Nirmala Rao, Jill P. Pell, Carlos Celis-Morales, Ian C. K. Wong, Frederick K. Ho, Patrick Ip

**Affiliations:** 1 Institute of Health and Wellbeing, University of Glasgow, Glasgow, United Kingdom; 2 Faculty of Medicine, Universidad Diego Portales, Santiago, Chile; 3 Institute of Cardiovascular and Medical Sciences, University of Glasgow, Glasgow, United Kingdom; 4 Faculty of Education, The University of Hong Kong, Hong Kong; 5 Centre of Exercise Physiology Research, Universidad Mayor, Providencia, Chile; 6 Laboratorio de Rendimiento Humano, Grupo de Estudio en Educación, Actividad Física y Salud, Universidad Católica del Maule, Talca, Chile; 7 Department of Pharmacology and Pharmacy, The University of Hong Kong, Hong Kong; 8 University College London School of Pharmacy, London, United Kingdom; 9 Department of Paediatrics and Adolescent Medicine, Queen Mary Hospital, The University of Hong Kong, Hong Kong

## Abstract

**Importance:**

Weight-for-height *z *score (WHZ) is a standard indicator of children’s nutritional status even though it does not fully reflect body fat.

**Objective:**

To examine the combined association of WHZ and body fat with early development in the East Asia and Pacific region.

**Design, Setting, and Participants:**

Children from the East Asia–Pacific Early Child Development Scales validation study, with full data available regarding their nutritional status and outcomes, were included in this cross-sectional analysis. In brief, a multilevel stratified random sampling was used to select representative samples from each participating country in the study. WHZ and body fat were independently trichotomized using established references and were combined to form a 9-category exposure variable. Data collection was performed between 2012 and 2014, and the analyses were conducted in June 2021.

**Main Outcomes and Measures:**

The binary outcome variable of not being developmentally on track (hereafter referred to as poor development) was defined as a score less than the 25th percentile in the following domains: cognitive, language, socioemotional, motor development, and total development score. Poisson regression models were used to analyze the associations between the combined categories and poor development, adjusted for sociodemographic factors.

**Results:**

A total of 6815 children (mean [SD] age, 4.02 [0.8] years; 3434 girls [50.4%]) had full data available and were included in this study. Compared with children with normal weight and normal fat, those with wasting and low body fat had the highest likelihood of total poor development (prevalence ratio, 1.47; 95% CI, 1.28-1.70), followed by those with normal weight but low fat (prevalence ratio, 1.23; 95% CI, 1.11-1.36). Similar associations were found in language, cognitive, and socioemotional development, but not in motor development.

**Conclusions and Relevance:**

Poor development was more commonly found in children with low body fat independent of WHZ (wasted or normal weight). Early public health strategies may consider using a combination of WHZ and body fat as an indicator of poor development.

## Key Points

**Question:**

What is the combined association of weight-for-height *z *score and body fat with early development in the East Asia and Pacific region?

**Findings:**

This cross-sectional study of 6815 children found that compared with children with normal weight and normal body fat, children with low body fat, either with wasting or normal weight, were more likely to have poor total, cognitive, language, and socioemotional development independent of confounding factors.

**Meaning:**

These findings reinforce the potential to use body fat, in addition to weight-for-height *z *score, to identify children who are not on track developmentally.

## Teaser

This cross-sectional study examines the combined association of weight-for-height *z *score and body fat with early childhood development in the East Asia and Pacific region.

## Introduction

Malnutrition can be measured in multiple ways. The World Health Organization (WHO) defines 3 types of malnutrition: undernutrition (including wasting [low weight-for-height], stunting [low height-for-age], and underweight [low weight-for-age]), micronutrient-related nutrition (inadequate intake of vitamins and minerals), and overweight (along with obesity and diet-related noncommunicable diseases).^1^ Despite efforts to reduce all forms of malnutrition, they are still prevalent among children. In 2020, approximately 149.2 million children were stunted, 45.4 million children were wasted, and 38.9 million children were overweight.^2^ These figures are particularly concerning in regions like Africa and Asia, where the highest proportion of stunted, wasted, and overweight children live.^2^

Weight-for-height *z *score (WHZ) is one of the easiest and least invasive indices used to assess children’s nutritional status and growth. A low WHZ often indicates acute undernutrition.^3^ However, although WHZ is strongly correlated with body fat levels,^3^ it does not reflect the relative proportions of fat-free mass and body fat mass. This distinction could be important in early childhood development (ECD) because fat plays a crucial role in children’s brain development and cerebral energy metabolism.^4,5^

Previous studies^6,7,8,9^ have independently reported the associations of nutritional status (mainly through WHZ) or body fat with suboptimal ECD; however, they investigated their associations separately,^6,7,8,9^ focusing on either nutritional status or body fat, not both.^9^ Despite the high correlation among these measurements, the accuracy of WHZ may vary according to the individual’s body fat.^3^ WHZ may also be a poor index in overweight children who have increased levels of fat-free mass. Consequently, investigating the combined association of these 2 anthropometric measurements with ECD could provide a better understanding of the association of different combinations of malnutrition and ECD. Considering that Asia is one of the regions with the highest prevalence of malnutrition (especially wasted and overweight children), this study aimed to explore the combined association of WHZ and body fat with not being developmentally on track or poor development considering total and 4 specific developmental domains, in children in the East Asia and Pacific Region.

## Methods

This cross-sectional study was approved by the human research ethics committee of the University of Hong Kong. Written informed consent was obtained from the parents of all participants. This study follows the Strengthening the Reporting of Observational Studies in Epidemiology (STROBE) reporting guideline for cross-sectional studies.^10^

The validation study for the East Asia–Pacific Early Child Development Scales (EAP-ECDS) was conducted in 6 countries in East Asia and Pacific Region, including Cambodia, China, Mongolia, Papua New Guinea, Timor-Leste, and Vanuatu, to create a common measurement tool to evaluate the development of children aged 3 to 5 years.^11,12^ In brief, multilevel stratified random sampling, including boys and girls residing in urban and rural settings, was used to select a representative sample from each participating country. The number of children assessed in each country ranged from 900 to 1803. The sampling plan was determined in collaboration with the National Census Department or National Statistical Institute in all countries except China. In China, data were collected from 5 provinces selected to represent a wide range of economic development levels (Guizhou, Heilongjiang, Jiangsu, Shanghai, and Zhejiang).^11,12^ Children with special educational needs and chronic medical conditions (including edema) were excluded from the study. Data collection was performed between 2012 and 2014, and the analyses for this study were conducted in June 2021.^11,12^ More information about the study can be found elsewhere.^11,12,13,14,15^

In this cross-sectional analysis, data from Timor-Leste were not included because of assessors’ concerns regarding the accuracy of the measurement of body weight. Some residential homes were in hilly regions where the assessor could not find a flat surface to measure body weight accurately. No similar feedback was received in other countries. As a result, the data from Timor-Leste were excluded from this analysis.

### Exposure

Body weight, height, mid–upper arm circumference (MUAC), and triceps skinfold thickness were measured by trained assessors following the protocols recommended by the WHO Multicenter Growth Reference Study.^16^ Training protocols can be found elsewhere.^13^ Body height, triceps skinfold thickness, and MUAC were measured to the nearest 1 mm, whereas weight was reported to the nearest 0.1 kg.

**Figure 1.**
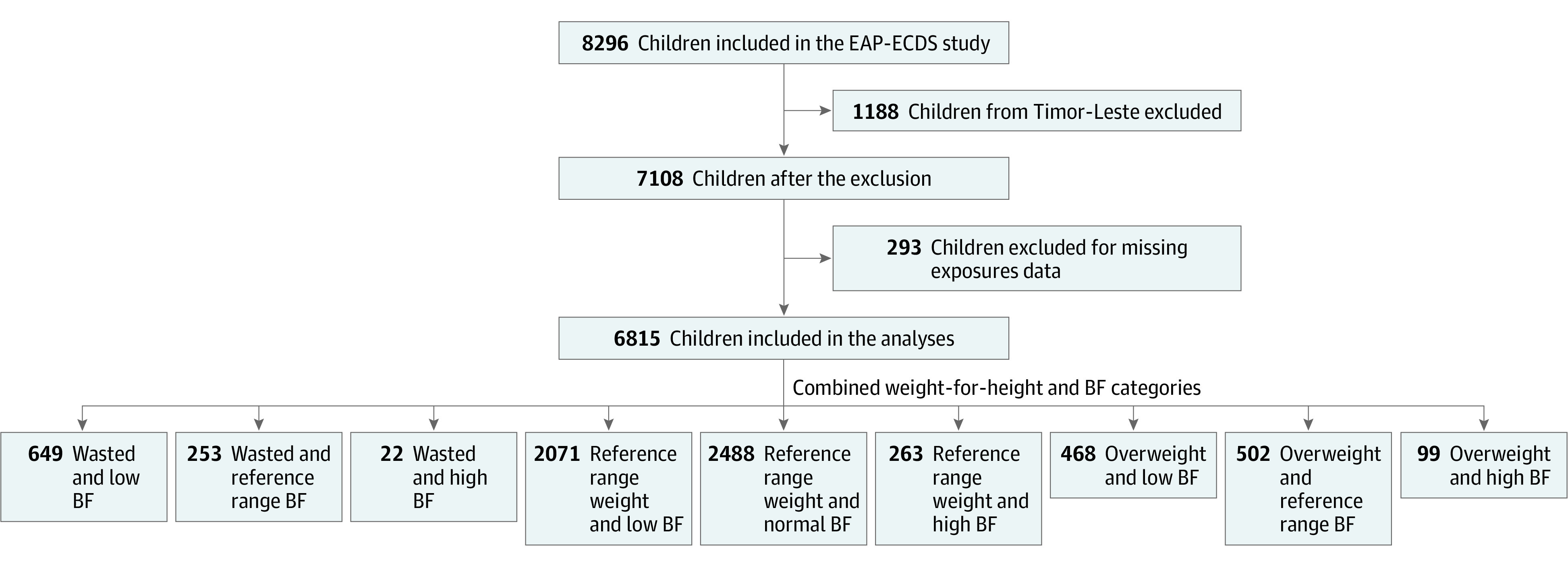
Flowchart of Children Included in the Study BF indicates body fat; EAP-ECDS, East Asia–Pacific Early Child Development Scales.

WHZ was standardized to sex-specific *z *scores. Applying the WHO Child Growth Standards,^16^ WHZ was classified as wasting (WHZ less than −2 SD), normal weight (WHZ greater than or equal to −2 and less than or equal to 2 SD), and overweight (WHZ greater than 2 SD). Body fat was classified using triceps skinfold thickness following the equations and age- and sex-specific cutoff points proposed by Frisancho^17^ in 1981: low body fat (less than 10th percentile), normal body fat (10th to 90th percentile), and high body fat (greater than 90th percentile). To investigate the combined association between WHZ and body fat, 9 categories were created: (1) wasted and low body fat, (2) wasted and normal body fat, (3) wasted and high body fat, (4) normal weight and low body fat, (5) normal weight and normal body fat, (6) normal weight and high body fat, (7) overweight and low body fat, (7) overweight and normal body fat, and (9) overweight and high body fat (Figure 1).

### Outcomes

The EAP-ECDS was administered to children, in individual sessions, by trained assessors. The original EAP-ECDS had 85 items that covered 7 domains of development: cognitive development (21 items); language and emergent literacy (henceforth referred to as language) (16 items); socioemotional development (15 items); motor development (7 items); health, hygiene, and safety (9 items); cultural knowledge and participation (10 items); and approaches to learning (7 items).^11^ Total development was estimated from the unweighted sum of the domain-specific scores. For this study, total development and 4 specific domains were selected because they were reported to be associated with WHZ and body fat previously: cognitive development, language development, socioemotional development, and motor development. For total and each specific domain, poor development was identified independently as a binary variable using a threshold of less than the 25th percentile in the population included in this analysis.

### Covariates

Age was calculated using dates of birth and date of assessment. Sociodemographic information was provided by caregivers in individual interviews.^11^ A socioeconomic status (SES) index was created to evaluate multidimensional family SES in this study. The SES index was the first eigenvalue of the principal component analysis results using the correlation matrix from paternal education level, maternal education level, and family assets, including electricity, radio, television, refrigerator, watch, mobile phone, bicycle, animal-drawn cart, agricultural land, livestock, and so forth. The method has been shown to be valid and reliable for representing the overall SES, as described elsewhere.^14,18^

### Statistical Analysis

Descriptive characteristics by the 9 categories are presented as means with SD for quantitative variables and as frequencies and percentages for categorical variables. Poisson regression models with robust SEs were used to analyze the cross-sectional associations of the combined combination of WHZ and body fat with poor development. The results are reported as prevalence ratio (PR) with their 95% CIs^19^ because this is a cross-sectional study that cannot be used to derive risk. Poisson regression models with robust SEs were used because they provide PR estimates that are relatively easy to interpret, instead of odds ratios.^20^ Robust SEs were used to correct for the underinflation when applying the Poisson model for binary outcomes.

Two models were run: model 1 was unadjusted, and model 2 was adjusted for age, sex, country of origin, urbanicity, and SES. Country of origin was modeled as a fixed factor because there were only 5 countries in this study and conditional independence should be achieved after including country as a fixed factor. In addition, 3 sensitivity analyses were performed: (1) poor development, identified as a score less than the age-specific 25th percentile (in a 1-year band); (2) body fat defined using fat-muscle area proportion (ie, MUAC) following the age- and sex-specific cutoff points of Frisancho^17^ instead of triceps skinfold thickness (available for 5200 children); and (3) body fat defined as triceps skinfold for age *z *score following the WHO recommendations (available for 4690 children aged ≤5 years). The first analysis was conducted because development is age dependent; the remaining 2 analyses were conducted to ensure the conclusions are robust against the methodological choice of body fat marker.

To investigate whether the associations between poor development and combined WHZ and body fat categories differed by subgroups, the analyses were rerun stratified by age category (at or older than the median age and younger than the median age [4.5 years]), sex (female and male), urbanicity (urban and rural), SES index score (at or above the median and less than the median [0.01]), and country of origin (East Asia [China and Mongolia], Southeast Asia [Cambodia], and the Pacific Region [Papua New Guinea and Vanuatu]). These subgroup variables were selected a priori on the basis of their strong influence on child growth and development. Only children with full data available were included in this study. All statistical analyses were performed using Stata statistical software version 16 (StataCorp). Two-sided *P* < .05 was considered significant.

## Results

From the original 7108 children, 6815 (95.9%; mean [SD] age, 4.02 [0.8] years; 3434 girls [50.4%]) had complete data on the exposure (combined WHZ and body fat categories), outcomes (total and 4 areas of development), and covariates and were included in this study (Figure 1). The children’s characteristics by combined WHZ and body fat categories are presented in Table 1. Overall, 4822 children (70.8%) had normal weight, 924 (13.5%) had wasting, and 1069 (15.7%) were overweight. In terms of body fat, 3188 (46.8%) had low body fat, 3243 (47.6%) had normal body fat, and 384 (5.6%) had high body fat. The most common combination was normal weight and normal body fat (2488 children [36.5%]), followed by normal weight and low body fat (2071 children [30.4%]), whereas wasting and high body fat was the least common (22 children [0.3%]). Girls tended to have low body fat, whereas boys had high body fat. Among the 99 children (1.4%) who were overweight and had high body fat, 51 (51.5%) lived in urban areas. Among the children with wasting and low body fat (649 children [9.5%]), 488 (75.2%) lived in Cambodia. Among children with normal weight and normal fat, 968 (39.0%) lived in Mongolia, and most of the overweight children with high body fat lived in China (67 children [67.6%]). Finally, children with low body fat, independently of WHZ, tended to come from a low SES family (Table 1).

**Table 1.** Characteristics of Participating Children by Nutritional Status^a^

Characteristic	Children, No. (%)									
	Overall	Wasted (WHZ less than −2)			Normal weight (WHZ −2 to 2)			Overweight (WHZ >2)		
		Low body fat	Normal body fat	High body fat	Low body fat	Normal body fat	High body fat	Low body fat	Normal body fat	High body fat
Total	6815 (100)	649 (9.5)	253 (3.7)	22 (0.3)	2071 (30.4)	2488 (36.5)	263 (3.9)	468 (6.9)	502 (7.4)	99 (1.4)
Age, mean (SD), y	4.02 (0.8)	3.94 (0.82)	4.28 (0.77)	4.18 (0.73)	3.96 (0.82)	4.08 (0.81)	3.96 (0.74)	3.89 (0.81)	4.05 (0.81)	3.82 (0.70)
Sex										
Female	3434 (50.4)	357 (55.0)	103 (40.7)	8 (36.4)	1206 (58.2)	1129 (45.4)	115 (43.7)	286 (61.1)	184 (36.6)	46 (46.5)
Male	3381 (49.6)	292 (45.0)	150 (59.3)	14 (63.6)	865 (41.8)	1359 (54.6)	148 (56.3)	182 (38.9)	318 (63.4)	53 (53.5)
Country										
China	1627 (23.9)	28 (4.3)	73 (28.9)	16 (72.7)	290 (14.0)	914 (36.7)	195 (74.1)	3 (0.6)	41 (8.1)	67 (67.6)
Cambodia	1490 (21.9)	488 (75.2)	17 (6.7)	0	879 (42.5)	73 (2.9)	0	27 (5.8)	6 (1.2)	0
Mongolia	1238 (18.2)	8 (1.2)	49 (19.4)	2 (9.1)	75 (3.6)	968 (39.0)	56 (21.3)	4 (0.8)	60 (12.0)	16 (16.2)
Papua New Guinea	1768 (25.9)	82 (12.7)	60 (23.7)	3 (13.6)	706 (34.1)	403 (16.2)	11 (4.2)	261 (55.8)	226 (45.0)	16 (16.2)
Vanuatu	692 (10.1)	43 (6.6)	54 (21.3)	1 (4.6)	121 (5.8)	130 (5.2)	1 (0.4)	173 (37.0)	169 (33.7)	0
Urbanicity										
Rural	4115 (60.4)	431 (66.4)	142 (56.1)	14 (63.6)	1230 (59.4)	1381 (55.5)	161 (61.2)	402 (85.9)	306 (61.0)	48 (48.5)
Urban	2700 (39.6)	218 (33.6)	111 (43.9)	8 (36.4)	841 (40.6)	1107 (44.5)	102 (38.8)	66 (14.1)	196 (39.0)	51 (51.5)
Composite socioeconomic *z *score, mean (SD)	0.13 (1.46)	−0.54 (1.25)	0.20 (1.40)	0.84 (1.42)	−0.32 (1.37)	0.82 (1.32)	1.31 (0.98)	−1.02 (1.02)	−0.41 (1.30)	1.02 (1.12)
WHZ, mean (SD)	0.09 (2.14)	−3.22 (0.93)	−3.38 (1.11)	−3.77 (1.29)	−0.26 (1.05)	0.07 (0.95)	0.36 (1.06)	3.66 (1.11)	3.64 (1.12)	3.06 (0.95)
Mid–upper arm circumference, mean (SD), mm	163.0 (28.6)	141.2 (30.2)	166.3 (25.4)	294.6 (29.6)	150.7 (29.6)	171.1 (18.5)	184.0 (20.4)	153.3 (21.8)	175.2 (26.1)	201.4 (25.6)
Triceps skinfold thickness, mean (SD), mm	7.58 (3.80)	4.00 (1.60)	8.80 (2.20)	17.6 (3.78)	4.76 (1.44)	0.33 (2.17)	16.9 (3.07)	5.29 (1.07)	8.65 (2.24)	17.7 (3.50)

Abbreviation: WHZ, weight-for-height *z *score.

^a^ Nutritional status was classified using WHZ following the World Health Organization recommendations. Body fat was classified using triceps skinfold thickness following the age- and sex-specific cutoff points of Frisancho (low, less than 10th percentile; normal, 10th to 90th percentile; high, greater than 90th percentile).^17^

**Figure 2.**
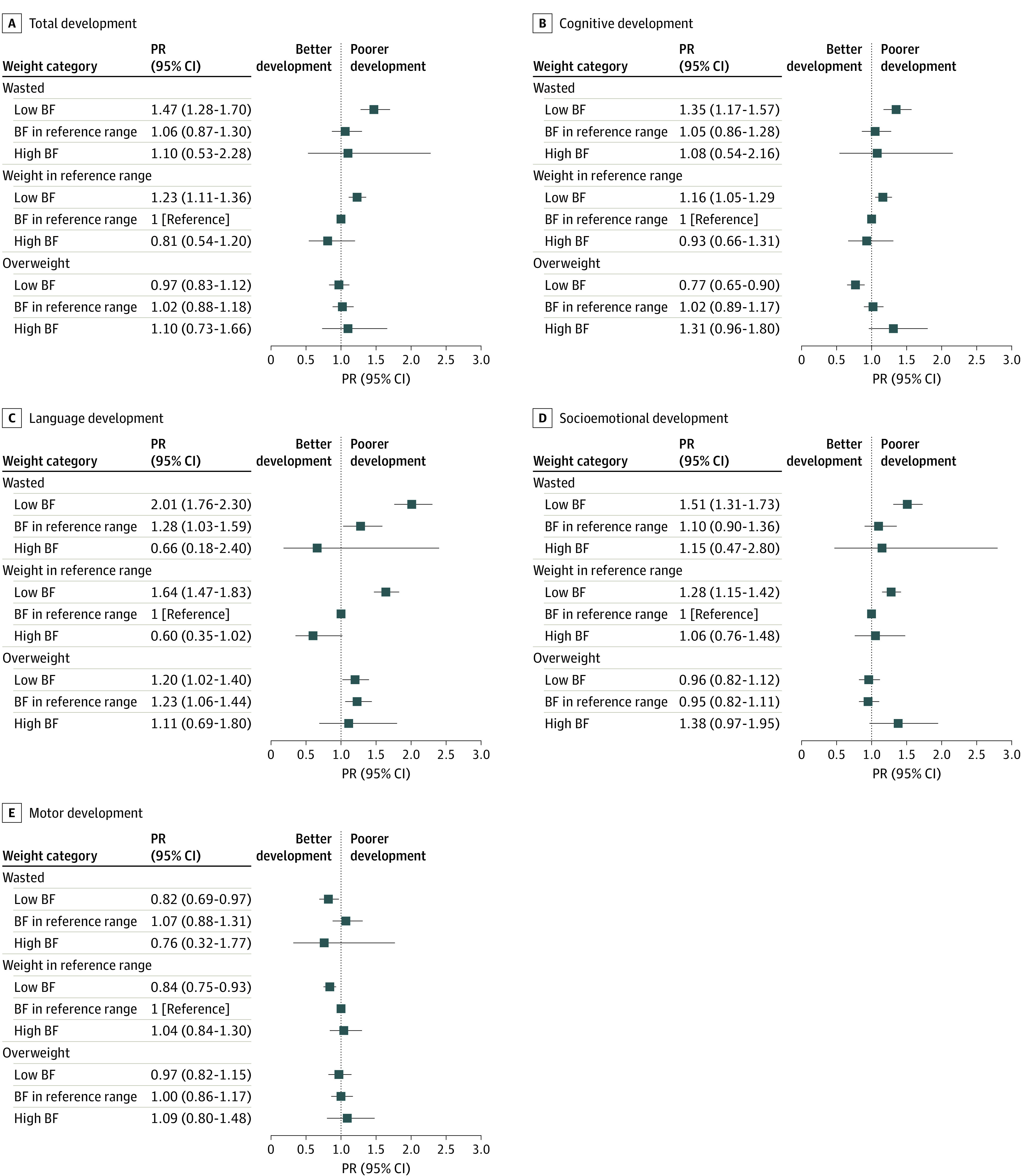
Combined Associations of Body Weight and Body Fat (BF) With Early Childhood Development Data are presented as prevalence ratio (PR) and 95% CIs for poor development (score below the 25th percentile). Analyses were adjusted by age, sex, socioeconomic status, urbanicity, and country of origin. There were 6815 participants in total. Nutritional status was classified using weight-for-height *z *score following the World Health Organization recommendations. BF was classified using triceps following the age- and sex-specific cutoff points of Frisancho^17^ (low, less than 10th percentile; normal, 10th to 90th percentile; high, greater than 90th percentile).

The associations between combined WHZ and body fat categories and poor domain-specific development are presented in Figure 1 and eTable 1 in the Supplement. In the unadjusted model (model 1), compared with children with normal weight and normal body fat, those with low body fat (independently of body weight) and with normal body fat (regardless of being wasted or overweight) had a higher likelihood of poor development in the total EAP-ECDS score. Similar patterns of association were found for cognitive, language, and socioemotional development domains (eTable 1 in the Supplement). The magnitude of these associations was attenuated when the analyses were further adjusted for the covariates (model 2). However, the associations were still significant for children with wasting and low body fat and for those with normal weight and low body fat, who had 1.47 (95% CI, 1.28-1.70) and 1.23 (95% CI, 1.11-1.36) times higher likelihood, respectively, of poor total development (Figure 2). Similar trends were identified for cognitive, language, and socioemotional development. For these 4 outcomes, children with wasting and low body fat were those with the highest likelihood of poor development (PR for total development, 1.47 [95% CI, 1.28-1.70]; PR for cognitive development, 1.35 [95% CI, 1.17-1.57]; PR for language development, 2.01 [95% CI, 1.76-2.30]; PR for socioemotional development, 1.51 [95% CI, 1.31-1.73]). For language development, children with overweight and low body fat or normal body fat also had 1.20 (95% CI, 1.02-1.40) and 1.23 (95% CI, 1.06-1.44) times, respectively, higher likelihood of poor development than those with normal weight and normal fat. Interestingly, children with low body fat and overweight and with low body fat (and either wasted or normal weight) had a lower likelihood of poor cognitive development and motor development, respectively, than children with normal weight and normal fat. No other significant associations were found for motor development (Figure 2). Similar results were observed when poor development was defined as a score less than the age-specific 25th percentile (data not shown).

Consistent findings were found even when body fat was defined using the fat-muscle area proportion (eTable 2 in the Supplement) or as *z *score for triceps skinfold for age using the WHO recommendations (eTable 3 in the Supplement). For instance, compared with children with normal weight and body fat, those with wasting and low body fat and those with normal weight and low body fat had 1.61 (95% CI, 1.38-1.88) and 1.26 (95% CI, 1.11-1.43) times higher likelihood of poor total development, respectively (eTable 2 in the Supplement). Similar results were found using the *z* score for triceps skinfold for age (PR for wasting and low body fat, 1.97 [95% CI, 1.66-2.33]; PR for normal weight and low body fat, 1.46 [95% CI, 1.29-1.66]) (eTable 3 in the Supplement).

Finally, significant interactions were identified for total development with age, sex, urbanicity, SES, and country in the subgroup analysis (Table 2). The interaction for the domain-specific outcomes of cognitive, language, socioemotional, and motor development can be found in eTable 4, eTable 5, eTable 6, eTable 7, and eTable 8 in the Supplement.

**Table 2.** Combined Associations of WHZ and Fat With Total Development by Moderators

Moderator	PR (95% CI)^a^								
	Wasted (WHZ less than −2)			Normal weight (WHZ −2 to 2)			Overweight (WHZ >2)		
	Low body fat	Normal body fat	High body fat	Low body fat	Normal body fat	High body fat	Low body fat	Normal body fat	High body fat
Age									
Less than the median	1.38 (1.14-1.67)	1.06 (0.79-1.43)	1.07 (0.65-1.78)	1.08 (0.93-1.24)	1 [Reference]	0.84 (0.53-1.34)	0.76 (0.61-0.95)	0.83 (0.66-1.04)	1.19 (0.76-1.85)
Median or older	1.57 (1.28-1.92)	1.10 (0.83-1.45)	1.09 (0.27-4.39)	1.41 (1.22-1.64)	1 [Reference]	0.68 (0.32-1.42)	1.31 (1.07-1.59)	1.21 (1.00-1.45)	0.74 (0.29-1.91)
* P* value for interaction	.36	.61	>.99	.003	NA	.44	<.001	.01	.29
Sex									
Female	1.72 (1.40-2.11)	1.04 (0.72-1.52)	NS	1.35 (1.15-1.59)	1 [Reference]	0.95 (0.48-1.86)	1.12 (0.90-1.38)	1.38 (1.11-1.71)	1.74 (1.12-2.70)
Male	1.31 (1.07-1.60)	1.06 (0.84-1.35)	1.22 (0.60-2.47)	1.17 (1.02-1.35)	1 [Reference]	0.72 (0.44-1.19)	0.87 (0.69-1.08)	0.86 (0.71-1.03)	0.68 (0.32-1.44)
* P* value for interaction	.03	.90	NS	.08	NA	.63	.04	<.001	.04
Urbanicity									
Rural	1.78 (1.50-2.12)	1.21 (0.91-1.1)	NS	1.55 (1.34-1.79)	1 [Reference]	0.60 (0.32-1.14)	1.26 (1.04-1.51)	1.19 (0.98-1.46)	1.20 (0.62-2.35)
Urban	1.25 (0.94-1.66)	0.92 (0.69-1.22)	1.66 (1.05-2.63)	0.97 (083-1.14)	1 [Reference]	1.02 (0.64-1.61)	0.66 (0.49;0.90)	0.76 (0.62-0.94)	0.89 (0.54-1.45)
* P* value for interaction	<.001	.84	NS	<.001	NA	.76	.96	.68	.28
Socioeconomic status									
Below the median	1.33 (1.14-1.54)	1.00 (0.79-1.26)	1.66 (0.88-3.14)	1.14 (1.01-1.28)	1 [Reference]	0.84 (0.46-1.51)	0.98 (0.84-1.14)	1.01 (0.87-1.18)	1.40 (0.96-2.04)
Median or higher	1.22 (0.78-1.89)	1.22 (0.82-1.82)	1.60 (0.37-6.96)	1.25 (0.99-1.56)	1 [Reference]	1.01 (0.60-1.68)	0.92 (0.55-1.52)	1.01 (0.73-1.40)	0.75 (0.29-1.95)
* P* value for interaction	.26	.05	.62	.42	NA	.57	.002	<.001	.13
Region of origin									
East Asia	1.12 (0.39-3.20)	1.27 (0.72-2.26)	0.80 (0.12-5.34)	0.40 (0.23-0.71)	1 [Reference]	0.60 (0.34-1.06)	NS	1.30 (0.73-2.40)	0.40 (0.13-1.25)
Southeast Asia	0.84 (0.52-1.38)	0.29 (0.04-1.92)	NS	0.83 (0.51-1.34)	1 [Reference]	NS	1.31 (0.73-2.35)	NS	NS
Pacific region	1.17 (0.97-1.41)	1.05 (0.86-1.27)	1.31 (0.81-2.13)	1.01 (0.90-1.13)	1 [Reference]	1.33 (0.90-1.94)	0.92 (0.84-1.10)	0.96 (0.84-1.10)	1.25 (0.86-1.83)
* P* value for interaction^b^	.78	.22	NS	.01	NA	NS	NS	NS	NS
* P* value for interaction^c^	.97	.87	.59	.006	NA	.02	NS	.25	.05

Abbreviations: NA, not applicable; NS, not sufficient numbers of children with poor development for moderator analysis; PR, prevalence ratio; WHZ, weight-for-height *z *score.

^a^ Data are PRs and 95% CIs for poor development (score <25th percentile). Analyses were adjusted by age, sex, socioeconomic status, urbanicity, and region of origin when these were not the moderators. There were a total of 6815 participants. Nutritional status was classified using WHZ following the World Health Organization recommendations. Fat was classified using triceps following the age- and sex-specific cutoff points of Frisancho^17^ (low, less than 10th percentile; normal, 10th to 90th percentile; high, greater than 90th percentile).

^b^ *P* values are for interactions between East Asia and Southeast Asia.

^c^ *P* values are for interactions between East Asia and the Pacific Region.

## Discussion

This cross-sectional study examined the combined association of WHZ and body fat with young children’s development in a large, representative sample of 3- to 5-year-olds using the EAP-ECDS validation study from 5 countries in the East Asia and Pacific Region.^11^ Compared with children with normal weight and normal fat, we found that those with low body fat, regardless of whether they had wasting or normal weight, were more likely to have poor total, cognitive, language, and socioemotional development. Poor development was most commonly found in children with wasting and low body fat, and Cambodia was the country with the highest prevalence of such children.

On the other hand, children with low body fat (and either wasting or normal weight) or low body fat and overweight were less likely to have poor motor development and cognitive development, respectively; however, this association may be related to unmeasured confounders. To our knowledge, this is the first study investigating the combined association of WHZ and body fat with poor development in children. Therefore, our study reinforces the importance of tackling malnutrition in low- and middle-income countries, as well as the potential to use body fat measurement, in addition to WHZ, to identify children who are not on track developmentally. Anthropometric parameters and EAP-ECDS domains were objectively measured by trained assessors and professionals with training in early childhood education, respectively, adding confidence to the measurement accuracy. Moreover, body fat was measured using skinfold thickness, one of the most suitable methods to measure body fat in children younger than 5 years.^21^

Childhood overweight and obesity are emerging in low- and middle-income countries. Current concerns about the global prevalence of overweight among children are based largely on WHZ, since fat and fat-free mass are rarely measured. A high body mass index or WHZ is frequently associated with high body fat; however, the accuracy of these indices depends on the individual’s body fat distribution, which could be affected by various factors.^3^ This is very relevant in children in whom most of the body fat is subcutaneous, those with small body sizes, or those in whom body fat is not homogeneously distributed. Our study demonstrated the relevance of measuring both WHZ and body fat in children, emphasizing the outcomes among stunted children as expected, but also in those who independently of their weight have low body fat. Because these children are often assessed by WHZ only, they represent a higher-risk group that is usually not the target in an intervention. This study also showed that children who were overweight but had low or normal body fat had a higher likelihood of language developmental problems. This coincides with previous evidence that obesity, the result of overnutrition, could lead to poorer development.^22^

The association between nutritional status and different development domains has been previously reported in children from different backgrounds using different anthropometric indices, but always in isolation.^6,7,23,24^ In Burkina Faso, Olsen et al^6^ used baseline data from 1608 children aged 6 to 23 months and found that children with moderately acute malnutrition showed worse language and motor development scores than those with better anthropometric measurements (MUAC, WHZ, height-for-age *z *score, and fat-free mass). In contrast with our results, those children with more body fat, measured using deuterium dilution techniques from total body water, had poorer scores.^6^ A prospective study^7^ of 455 Ethiopian children demonstrated that fat mass at birth was associated with a higher global development score at 1 and 5 years in the unadjusted model. After adjusting for confounder factors, the magnitude of the associations disappeared.^7^ In the same cohort, Abera et al^8^ identified that for each 1-kg increase in fat mass tissue since birth, there was a 5.7-point increment in the Strengths and Difficulties Questionnaire score at 5 years, in which a higher score was associated with a higher likelihood of mental health problems. In comparison with our study, these studies explored WHZ or body fat association individually with poor development using different development domains. The latter emphasizes our findings’ novelty, providing a more comprehensive overview of nutrition associated with childhood development and filling gaps in the existing evidence. In addition, our results also highlight the urgency of a more thorough evaluation of children’s nutritional status, which moves beyond body weight or body height to identify risk because we found that children with normal weight but low body fat were at higher risk of poor development.

Despite increasing understanding of the role of nutrition on ECD, the elimination of malnutrition in low- and middle-income countries is still challenging. Poverty and food insecurity (for undernutrition), as well as westernized diets (for obesity), are among the possible major causes.^25^ Early nutrition support and interventions reducing risk factors or promoting protective factors are promising strategies to promote optimal development throughout the life cycle, especially in countries with a high prevalence of malnutrition, including under- and overnutrition.

### Limitations

The findings of this study need to be interpreted with the following caveats. First, although arm anthropometry, through the measurement of MUAC and triceps skinfold thickness, has shown to be a good proxy and correlates strongly with fat mass assessing body composition in children, its correlation with fat-free mass is weaker.^26^ Second, because of the cross-sectional nature of this study, causality cannot be inferred. Nonetheless, body fat appears to be a useful risk marker that helps identify children with poor development. Future prospective cohort studies should investigate the prospective association between WHZ and body fat to development and also explore the potential mechanisms involved. Third, some potential confounders, such as dietary factors and physical activity levels, were not included. The latter could be one of the reasons why some children with low body fat were found to have better motor or cognitive development. However, these factors are likely to be downstream of deprivation, for which we already adjusted. Fourth, body fat categories were defined using the formulas calculated by Frisancho^17^ in 1981 for US children, which means the cutoff points used might not be entirely applicable for children in the East Asia and Pacific Region. However, the sensitivity analyses using the WHO references did not alter our conclusions.

## Conclusions

In conclusion, poor development was more commonly found in children with low body fat whether they had wasting or normal weight. Our study reinforces the potential to use body fat, in addition to WHZ, to identify children who are not developmentally on track.

## Notes

Supplement. **eTable 1.** Combination of Nutritional Status and Triceps Categories **eTable 2.** Combination of Nutritional Status and Fat-Muscle Area Proportion **eTable 3.** Combination of Nutritional Status and Triceps Categories Using WHO *z* Score **eTable 4.** Combined Associations of WHZ and Fat With Early Childhood Development by Age **eTable 5.** Combined Associations of WHZ and Fat With Early Childhood Development by Sex **eTable 6.** Combined Associations of WHZ and Fat With Early Childhood Development by Urbanicity **eTable 7.** Combined Associations of WHZ and Fat With Early Childhood Development by SES **eTable 8.** Combined Associations of WHZ and Fat With Early Childhood Development by Country of Origin
