# Supplementary material for: Weight-for-Height, Body Fat, and Development in Children in the East Asia and Pacific Region
Source: JAMA Netw Open. 2022 Jan 6;5(1):e2142458. doi: 10.1001/jamanetworkopen.2021.42458 (PMC8739761; doi:10.1001/jamanetworkopen.2021.42458)
Supplement: Supplement. — eTable 1. Combination of Nutritional Status and Triceps Categories eTable 2. Combination of Nutritional Status and Fat-Muscle Area Proportion eTable 3. Combination of Nutritional Status and Triceps Categories Using WHO z Score eTable 4. Combined Associations of WHZ and Fat With Early Childhood Development by Age eTable 5. Combined Associations of WHZ and Fat With Early Childhood Development by Sex eTable 6. Combined Associations of WHZ and Fat With Early Childhood Development by Urbanicity eTable 7. Combined Associations of WHZ and Fat With Early Childhood Development by SES eTable 8. Combined Associations of WHZ and Fat With Early Childhood Development by Country of Origin [file jamanetwopen-e2142458-s001.pdf]

Supplemental Online Content

Petermann-Rocha F, Rao N, Pell JP, et al. Weight-for-height, body fat, and development in children in the East Asia and Pacific region. *JAMA Netw Open*. 2022;5(1):e2142458. doi:10.1001/jamanetworkopen.2021.42458

eTable 1. Combination of Nutritional Status and Triceps Categories

eTable 2. Combination of Nutritional Status and Fat-Muscle Area Proportion

eTable 3. Combination of Nutritional Status and Triceps Categories Using WHO *z* Score

eTable 4. Combined Associations of WHZ and Fat With Early Childhood Development by Age

eTable 5. Combined Associations of WHZ and Fat With Early Childhood Development by Sex

eTable 6. Combined Associations of WHZ and Fat With Early Childhood Development by Urbanicity

eTable 7. Combined Associations of WHZ and Fat With Early Childhood Development by SES

eTable 8. Combined Associations of WHZ and Fat With Early Childhood Development by Country of Origin

This supplemental material has been provided by the authors to give readers additional information about their work.

© 2022 Petermann-Rocha F et al. *JAMA Network Open.*

eTable 1. Combination of Nutritional Status and Triceps Categories

		Model 1		
	Exposures	Wasted	Normal	Overweight
Outcome		PR (95% CI)	PR (95% CI)	PR (95% CI)
**Total development**	**Low body fat**	1.76 (1.53; 2.04)	1.70 (1.52; 1.90)	2.34 (2.03; 2.69)
	**Normal body fat**	1.61 (1.29; 2.00)	1.00 (Ref.)	2.25 (1.95; 2.58)
	**High body fat**	1.06 (0.43; 2.58)	0.44 (0.29; 0.68)	0.82 (0.50; 1.34)
**Cognitive development**	**Low body fat**	1.51 (1.31; 1.75)	1.52 (1.37; 1.69)	1.72 (1.47; 2.00)
	**Normal body fat**	1.51 (1.23; 1.87)	1.00 (Ref.)	2.09 (1.83; 2.39)
	**High body fat**	0.95 (0.39; 2.32)	0.54 (0.37; 0.78)	1.01 (0.67; 1.52)
**Language development**	**Low body fat**	2.79 (2.43; 3.21)	2.45 (2.18; 2.75)	2.94 (2.53; 3.41)
	**Normal body fat**	1.92 (1.52; 2.42)	1.00 (Ref.)	2.65 (2.28; 3.09)
	**High body fat**	0.67 (0.18; 2.52)	0.34 (0.19; 0.59)	0.82 (0.46; 1.44)
**Socioemotional development**	**Low body fat**	1.88 (1.64; 2.17)	1.77 (1.59; 1.97)	2.27 (1.97; 2.61)
	**Normal body fat**	1.57 (1.26; 1.96)	1.00 (Ref.)	1.94 (1.67; 2.26)
	**High body fat**	1.05 (0.43; 2.56)	0.64 (0.45; 0.91)	1.05 (0.68; 1.61)
**Motor development**	**Low body fat**	0.78 (0.66; 0.92)	0.85 (0.77; 0.94)	1.03 (0.88; 1.21)
	**Normal body fat**	1.10 (0.90; 1.34)	1.00 (Ref.)	1.11 (0.96; 1.29)
	**High body fat**	0.67 (0.28; 1.64)	0.94 (0.76; 1.17)	1.08 (0.79; 1.48)

Data presented as prevalence ratio (PR) and 95% CIs for poor development (score <25th centile). Analyses were unadjusted. Total participants: 6 815.
Nutritional status was classified using weight-for-height (z-score) following the WHO recommendations. Fat was classified using triceps following the age- and sex-specific cut-off points of Frisancho 1981 (Normal: P10-P90; low <P10; High >P90).

© 2022 Petermann-Rocha F et al. *JAMA Network Open.*

eTable 2. Combination of Nutritional Status and Fat-Muscle Area Proportion

		Model 1			Model 2		
		Wasted	Normal	Overweight	Wasted	Normal	Overweight
Outcome	Exposures	PR (95% CI)	PR (95% CI)	PR (95% CI)	PR (95% CI)	PR (95% CI)	PR (95% CI)
**Total development**	**Low body fat**	2.14 (1.82; 2.52)	2.02 (1.77; 2.31)	3.06 (2.49; 3.77)	1.61 (1.38; 1.88)	1.26 (1.11; 1.43)	0.97 (0.77; 1.23)
	**Normal body fat**	1.92 (1.41; 2.62)	1.00 (Ref.)	2.80 (2.28; 3.44)	1.22 (0.93; 1.60)	1.00 (Ref.)	1.16 (0.94; 1.42)
	**High body fat**	1.44 (0.60; 3.49)	0.50 (0.31; 0.80)	0.67 (0.37; 1.22)	1.24 (0.76; 2.02)	0.70 (0.45; 1.07)	0.80 (0.48; 1.33)
**Cognitive development**	**Low body fat**	1.71 (1.46; 2.01)	1.84 (1.63; 2.08)	2.49 (2.02; 3.08)	1.47 (1.25; 1.72)	1.27 (1.13; 1.43)	0.90 (0.72; 1.13)
	**Normal body fat**	1.98 (1.51; 2.60)	1.00 (Ref.)	2.75 (2.29; 3.30)	1.33 (1.03; 1.71)	1.00 (Ref.)	1.22 (1.03; 1.46)
	**High body fat**	0.92 (0.32; 2.63)	0.58 (0.39; 0.86)	0.97 (0.62; 1.51)	0.84 (0.37; 1.90)	0.81 (0.56; 1.19)	1.20 (0.85; 1.68)
**Language development**	**Low body fat**	4.46 (3.75; 5.31)	3.82 (3.27; 4.47)	5.32 (4.28; 6.61)	2.63 (2.22; 3.11)	2.07 (1.78; 2.41)	1.53 (1.21; 1.95)
	**Normal body fat**	2.51 (1.75; 3.61)	1.00 (Ref.)	3.56 (2.77; 4.58)	1.58 (1.16; 2.17)	1.00 (Ref.)	1.47 (1.14; 1.91)
	**High body fat**	2.89 (1.33; 6.25)	0.52 (0.29; 0.94)	0.86 (0.43; 1.69)	2.43 (1.71; 3.46)	0.69 (0.40; 1.20)	0.97 (0.54; 1.74)
**Socioemotional development**	**Low body fat**	2.26 (1.92; 2.64)	2.09 (1.83; 2.38)	3.05 (2.48; 3.75)	1.67 (1.43; 1.95)	1.34 (1.18; 1.52)	1.05 (0.83; 1.32)
	**Normal body fat**	1.83 (1.33; 2.51)	1.00 (Ref.)	2.54 (2.05; 3.15)	1.29 (0.97; 1.71)	1.00 (Ref.)	1.20 (0.96; 1.50)
	**High body fat**	1.42 (0.59; 3.43)	0.69 (0.47; 1.03)	0.92 (0.56; 1.52)	1.35 (0.78; 2.32)	0.91 (0.63; 1.32)	1.11 (0.73; 1.70)
**Motor development**	**Low body fat**	0.78 (0.65; 0.92)	0.80 (0.72; 0.90)	1.08 (0.85; 1.39)	0.84 (0.70; 1.01)	0.82 (0.73; 0.93)	1.04 (0.80; 1.34)
	**Normal body fat**	1.41 (1.12; 1.78)	1.00 (Ref.)	1.24 (1.01; 1.52)	1.33 (1.05; 1.68)	1.00 (Ref.)	1.04 (0.84; 1.27)
	**High body fat**	0.73 (0.30; 1.76)	0.94 (0.75; 1.18)	0.95 (0.68; 1.31)	0.76 (0.33; 1.75)	1.03 (0.82; 1.29)	0.99 (0.72; 1.36)

Data presented as prevalence ratio (PR) and 95% CIs for poor development (score <25th centile). Analyses were adjusted by age, sex, SES, urbanicity and country of origin. Total participants: 5 200. Nutritional status was classified using weight-for-height (z-score) following the WHO recommendations. Fat was classified using upper arm fat area following the age- and sex-specific cut-off points of Frisancho 1981 (Normal: P10-P90; low <P10; High >P90).

© 2022 Petermann-Rocha F et al. *JAMA Network Open.*

eTable 3. Combination of Nutritional Status and Triceps Categories Using WHO *z* Score

Outcome	Exposures	Model 1 Wasted PR (95% CI)	Model 1 Normal PR (95% CI)	Model 1 Overweight PR (95% CI)	Model 2 Wasted PR (95% CI)	Model 2 Normal PR (95% CI)	Model 2 Overweight PR (95% CI)
**Total development**	**Low body fat**	1.87 (1.58; 2.22)	1.86 (1.63; 2.11)	2.13 (1.70; 2.65)	1.97 (1.66; 2.33)	1.46 (1.29; 1.66)	1.04 (0.82; 1.32)
	**Normal body fat**	1.56 (1.26; 1.92)	1.00 (Ref.)	1.94 (1.68; 2.24)	1.02 (0.83; 1.26)	1.00 (Ref.)	0.90 (0.77; 1.04)
	**High body fat**	1.16 (0.42; 3.16)	0.69 (0.49; 0.97)	1.00 (0.64; 1.54)	1.02 (0.47; 2.19)	1.18 (0.86; 1.62)	1.22 (0.83; 1.80)
**Cognitive development**	**Low body fat**	1.31 (1.08; 1.58(	1.57 (1.38; 1.79)	1.46 (1.12; 1.90)	1.36 (1.11; 1.65)	1.28 (1.12; 1.45)	0.79 (0.60; 1.04)
	**Normal body fat**	1.45 (1.18; 1.77)	1.00 (Ref.)	1.70 (1.48; 1.96)	1.03 (0.84; 1.26)	1.00 (Ref.)	0.88 (0.76; 1.02)
	**High body fat**	1.03 (0.38; 2.83)	0.74 (0.54; 1.01)	1.15 (0.79; 1.68)	1.04 (0.49; 2.20)	1.15 (0.86; 1.54)	1.39 (1.03; 1.87)
**Language development**	**Low body fat**	2.86 (2.46; 3.33)	2.61 (2.30; 2.96)	2.45 (1.95; 3.08)	2.39 (2.04; 2.79)	1.83 (1.62; 2.08)	1.11 (0.86; 1.42)
	**Normal body fat**	1.83 (1.48; 2.26)	1.00 (Ref.)	2.04 (1.75; 2.39)	1.23 (1.00; 1.51)	1.00 (Ref.)	1.00 (0.84; 1.17)
	**High body fat**	0.90 (0.25; 3.28)	0.40 (0.24; 0.66)	1.10 (0.70; 1.74)	0.75 (0.20; 2.72)	0.96 (0.43; 1.11)	1.41 (0.93; 2.13)
**Socioemotional development**	**Low body fat**	1.80 (1.53; 2.13)	1.85 (1.64; 2.09)	2.27 (1.86; 1.08)	1.77 (1.50; 2.08)	1.44 (1.28; 1.63)	1.12 (0.91; 1.39)
	**Normal body fat**	1.53 (1.25; 1.87)	1.00 (Ref.)	1.60 (1.37; 1.85)	1.06 (0.86; 1.30)	1.00 (Ref.)	0.79 (0.68; 0.93)
	**High body fat**	0.72 (0.20; 2.60)	0.79 (0.58; 1.08)	1.20 (0.83; 1.75)	0.71 (0.21; 2.39)	1.25 (0.93; 1.68)	1.50 (1.08; 2.09)
**Motor development**	**Low body fat**	0.88 (0.72; 1.07)	0.86 (0.75; 0.99)	0.88 (0.65; 1.19)	0.97 (0.79; 1.19)	0.90 (0.78; 1.05)	0.95 (0.70; 1.30)
	**Normal body fat**	1.00 (0.81; 1.24)	1.00 (Ref.)	1.10 (0.95; 1.28)	1.00 (0.80; 1.23)	1.00 (Ref.)	1.08 (0.92; 1.27)
	**High body fat**	1.05 (0.46; 2.41)	1.03 (0.83; 1.28)	1.16 (0.85; 1.58)	1.15 (0.52; 2.54)	1.07 (0.86; 1.33)	1.13 (0.83; 1.53)

Data presented as prevalence ratio (PR) and 95% CIs for poor development (score <25th centile). Analyses were adjusted by age, sex, SES, urbanicity and country of origin. Total participants: 4 690. Nutritional status and triceps skinfold were classified using weight-for-height (z-score) and triceps skinfold-for-age (z-score), respectively, following the WHO recommendations.

© 2022 Petermann-Rocha F et al. *JAMA Network Open.*

eTable 4. Combined Associations of WHZ and Fat With Early Childhood Development by Age

	Wasted (WHZ < -2 SD)			Normal weight (-2 ≤ WHZ ≤ 2 SD)			Overweight (WHZ > 2 SD)		
	Low body fat	Normal body fat	High body fat	Low body fat	Normal body fat	High body fat	Low body fat	Normal body fat	High body fat
	PR (95% CI)	PR (95% CI)	PR (95% CI)	PR (95% CI)	PR (95% CI)	PR (95% CI)	PR (95% CI)	PR (95% CI)	PR (95% CI)
**Cognitive development**									
< median	1.06 (0.86; 1.31)	0.98 (0.71; 1.35)	1.07 (0.56; 2.03)	0.96 (0.83; 1.11)	1.00 (Ref.)	0.96 (0.65; 1.44)	0.62 (0.49; 079)	0.97 (0.79; 1.18)	1.27 (0.88; 1.82)
≥ median	1.72 (1.41; 2.10)	1.14 (0.88; 1.48)	1.06 (0.29; 3.90)	1.42 (1.23; 1.63)	1.00 (Ref.)	072 (0.36; 1.43)	1.01 (0.82; 1.25)	1.08 (0.90; 1.29)	1.18 (0.62; 2.18)
$P_{interaction}$	.006	.14	.99	<.001		.19	<.001	.009	.56
**Language development**									
< median	2.04 (1.69; 2.46)	1.36 (0.98; 1.88)	0.68 (0.12; 3.89)	1.52 (1.29; 1.78)	1.00 (Ref.)	0.72 (0.39; 1.33)	0.89 (0.70; 1.13)	1.00 (0.77; 1.29)	1.41 (0.86; 2.30)
≥ median	1.88 (1.54; 2.29)	1.25 (0.94; 1.66)	0.60 (0.09; 4.14)	1.75 (1.50; 2.03)	1.00 (Ref.)	0.37 (0.12; 1.13)	1.64 (1.35; 1.99)	1.41 (1.16; 1.71)	0.55 (0.16; 1.90)
$P_{interaction}$	.13	.94	.89	.39		.216	<.001	.003	.12
**Socioemotional development**									
< median	1.36 (1.12; 1.64)	1.11 (0.82; 1.51)	0.58 (0.11; 3.02)	1.15 (0.99; 1.33)	1.00 (Ref.)	1.09 (0.74; 1.61)	0.81 (0.66; 1.01)	0.85 (0.68; 1.07)	1.54 (1.08; 2.18)
≥ median	1.71 (1.40; 2.08)	1.14 (0.86; 1.52)	1.72 (0.53; 5.60)	1.43 (1.23; 1.67)	1.00 (Ref.)	0.88 (0.46; 1.68)	1.24 (1.01; 1.53)	1.07 (0.87; 1.32)	075 (0.27; 2.09)
$P_{interaction}$	.06	.50	.27	.005		.30	<.001	.008	.14
**Motor development**									
< median	0.77 (0.61; 0.97)	1.09 (0.80; 1.49)	1.18 (0.49; 2.82)	0.78 (0.68; 0.91)	1.00 (Ref.)	0.82 (0.60; 1.12)	0.87 (0.68; 1.12)	1.06 (0.86; 1.31)	1.09 (0.75; 1.57)
≥ median	0.88 (0.69; 1.13)	1.08 (0.84; 1.40)	0.36 (0.06; 2.35)	0.91 (0.78; 1.06)	1.00 (Ref.)	1.37 (1.00; 1.87)	1.18 (0.93; 1.48)	0.97 (0.78; 1.21)	1.07 (0.61; 1.89)
$P_{interaction}$	.51	.70	.22	.08		.10	.001	.49	.73

Data presented as prevalence ratio (PR) and 95% CIs for poor development (score <25th centile). Analyses were adjusted by sex, SES, urbanicity and country of origin. Total participants: 6 815. Nutritional status was classified using weight-for-height (z-score) following the WHO recommendations. Fat was classified using triceps following the age- and sex-specific cut-off points of Frisancho 1981 (Normal: P10-P90; low <P10; High >P90). SD: standard deviations.

© 2022 Petermann-Rocha F et al. *JAMA Network Open.*

eTable 5. Combined Associations of WHZ and Fat With Early Childhood Development by Sex

	Wasted (WHZ < -2 SD)			Normal weight (-2 ≤ WHZ ≤ 2 SD)			Overweight (WHZ > 2 SD)		
	**Low body fat**	**Normal body fat**	**High body fat**	**Low body fat**	**Normal body fat**	**High body fat**	**Low body fat**	**Normal body fat**	**High body fat**
	**PR (95% CI)**	**PR (95% CI)**	**PR (95% CI)**	**PR (95% CI)**	**PR (95% CI)**	**PR (95% CI)**	**PR (95% CI)**	**PR (95% CI)**	**PR (95% CI)**
**Cognitive development**									
Female	1.44 (1.18; 1.76)	1.12 (0.81; 1.54)	NS	1.20 (1.04; 1.40)	1.00 (Ref.)	0.97 (0.56; 1.66)	0.83 (0.67; 1.03)	1.16 (0.94; 1.42)	1.62 (1.12; 2.34)
Male	1.28 (1.03; 1.59)	1.01 (0.78; 1.31)	1.28 (0.64; 2.54)	1.15 (0.99; 1.33)	1.00 (Ref.)	0.90 (0.57; 1.40)	0.72 (0.56; 0.92)	0.95 (0.80; 1.14)	1.05 (0.62; 1.79)
$P_{interaction}$	.24	.53	NS	.37		.90	.24	.07	.18
**Language development**									
Female	2.34 (1.90; 2.90)	1.41 (0.94; 2.09)	NS	1.91 (1.59; 2.30)	1.00 (Ref.)	0.60 (0.23; 1.56)	1.44 (1.13; 1.83)	1.90 (1.48; 2.43)	2.17 (1.33; 3.53)
Male	1.86 (0.56; 2.23)	1.21 (0.94; 1.56)	0.71 (0.19; 2.63)	1.52 (1.32; 1.76)	1.00 (Ref.)	0.59 (0.31; 1.13	1.09 (0.88; 1.36)	0.97 (0.79; 1.18)	0.50 (0.18; 1.36)
$P_{interaction}$	.03	.50	NS	.02		>.99	.06	<.001	.009
**Socioemotional development**									
Female	1.54 (1.27; 1.88)	1.08 (0.76; 1.55)	NS	1.24 (1.06; 1.45)	1.00 (Ref.)	1.41 (0.90; 2.21)	0.94; 0.76; 1.17)	1.15 (0.91; 1.44)	2.06 (1.42; 2.98)
Male	1.48 (1.22; 1.81)	1.12 (0.87; 1.44)	1.39 (0.58; 3.33)	1.34 (1.16; 1.55)	1.00 (Ref.)	0.82 (0.50; 1.34)	0.99 (079; 1.25)	0.86 (0.70; 1.05)	0.83 (0.42; 1.62)
$P_{interaction}$	.33	.90	NS	.94		.17	.66	.01	.02
**Motor development**									
Female	0.92 (0.70; 1.14)	1.19 (0.89; 1.59)	1.18 (0.39; 3.54)	0.92 (0.79; 1.06)	1.00 (Ref.)	1.09 (0.78; 1.52)	1.03 (0.82; 1.29)	1.10 (0.88;1.40)	1.06 (0.68; 1.64)
Male	0.71 (0.55; 0.93)	0.99 (0.75; 1.30)	0.55 (0.16; 1.91)	0.75 (0.63; 0.88)	1.00 (Ref.)	1.00 (0.75; 1.34)	0.89 (0.67; 1.18)	0.93 (0.76; 1.15)	1.12 (0.73; 1.71)
$P_{interaction}$	.13	.27	.37	.05		.85	.22	.15	.74

Data presented as prevalence ratio (PR) and 95% CIs for poor development (score <25th centile). Analyses were adjusted by age, SES, urbanicity and country of origin. Total participants: 6 815. Nutritional status was classified using weight-for-height (z-score) following the WHO recommendations. Fat was classified using triceps following the age- and sex-specific cut-off points of Frisancho 1981 (Normal: P10-P90; low <P10; High >P90).

NS: Not sufficient poor developed children for moderator analysis. SD: standard deviations.

© 2022 Petermann-Rocha F et al. *JAMA Network Open.*

eTable 6. Combined Associations of WHZ and Fat With Early Childhood Development by Urbanicity

	Wasted (WHZ < -2 SD)			Normal weight (-2 ≤ WHZ ≤ 2 SD)			Overweight (WHZ > 2 SD)		
	**Low body fat**	**Normal body fat**	**High body fat**	**Low body fat**	**Normal body fat**	**High body fat**	**Low body fat**	**Normal body fat**	**High body fat**
	**PR (95% CI)**	**PR (95% CI)**	**PR (95% CI)**	**PR (95% CI)**	**PR (95% CI)**	**PR (95% CI)**	**PR (95% CI)**	**PR (95% CI)**	**PR (95% CI)**
**Cognitive development**									
Rural	1.61 (1.35; 1.93)	1.08 (0.80; 1.45)	NS	1.48 (1.25; 1.70)	1.00 (Ref.)	0.65 (0.38; 1.12)	0.92 (0.75; 1.12)	1.02 (0.84; 1.25)	1.34 (0.77; 2.34)
Urban	1.23 (0.92; 1.62)	1.02 (0.79; 1.33)	1.72 (1.12; 2.66)	0.92 (0.79; 1.08)	1.00 (Ref.)	1.25 (0.81; 1.94)	0.75 (0.57; 0.99)	0.96 (0.80; 1.15)	1.14 (0.81; 1.61)
$P_{interaction}$	.01	.34	NS	<.001		.45	.01	.001	.37
**Language development**									
Rural	2.44 (2.08; 2.87)	1.46 (1.10; 1.95)	NS	2.04 (1.76; 2.36)	1.00 (Ref.)	0.38 (0.16; 0.90)	1.52 (1.26; 1.84)	1.41 (1.14; 1.73)	0.67 (0.24; 1.83)
Urban	1.57 (1.14; 2.15)	1.05 (0.77; 1.44)	1.04 (0.33; 3.31)	1.29 (1.07; 1.54)	1.00 (Ref.)	0.92 (0.51; 1.66)	0.88 (0.63; 1.22)	0.93 (0.73; 1.18)	1.24 (0.74; 2.06)
$P_{interaction}$	<.001	.65	NS	<.001		.39	.64	.54	.43
**Socioemotional development**									
Rural	1.0 (1.45; 2.00)	1.28 (0.98; 1.66)	0.56 (0.08; 3.79)	1.51 (1.32; 1.2)	1.00 (Ref.)	1.02 (0.66; 1.57)	1.18 (0.98; 1.41)	0.94 (0.76; 1.16)	1.19 (0.64; 2.20)
Urban	1.41 (1.06; 1.89)	0.90 (0.65; 1.24)	1.48 (0.58; 3.79)	1.06 (0.88; 1.27)	1.00 (Ref.)	1.04 (0.62; 1.73)	0.64 (0.45; 0.92)	0.91 (0.72; 1.14)	1.37 (0.93; 2.02)
p-interaction	0.006	.42	.38	.001		.42	.54	.01	.95
**Motor development**									
Rural	0.92 (0.73; 1.15)	1.11 (0.81; 1.52)	0.36 (0.05; 2.44)	0.93 (0.79; 1.10)	1.00 (Ref.)	1.24 (0.93; 1.66)	1.06 (0.85; 1.33)	1.12 (0.89; 1.41)	1.21 (0.73; 2.01)
Urban	0.75 (0.58; 0.6)	1.04 (0.81; 1.34)	1.12 (0.45; 2.79)	0.79 (0.68; 0.91)	1.00 (Ref.)	0.82 (0.58; 1.15)	0.99 (0.74; 1.34)	0.91 (0.74; 1.13)	1.00 (0.69; 1.46)
$P_{interaction}$	.17	.97	.32	.11		.02	.65	.67	.44

Data presented as prevalence ratio (PR) and 95% CIs for poor development (score <25th centile). Analyses were adjusted by age, sex, SES and country of origin. Total participants: 6 815. Nutritional status was classified using weight-for-height (z-score) following the WHO recommendations. Fat was classified using triceps following the age- and sex-specific cut-off points of Frisancho 1981 (Normal: P10-P90; low <P10; High >P90).
NS: Not sufficient poor developed children for moderator analysis. SD: standard deviations.

© 2022 Petermann-Rocha F et al. *JAMA Network Open.*

eTable 7. Combined Associations of WHZ and Fat With Early Childhood Development by SES

	Wasted (WHZ < -2 SD)			Normal weight (-2 ≤ WHZ ≤ 2 SD)			Overweight (WHZ > 2 SD)		
	**Low body fat**	**Normal body fat**	**High body fat**	**Low body fat**	**Normal body fat**	**High body fat**	**Low body fat**	**Normal body fat**	**Overweight & TS >90th centile**
	**PR (95% CI)**	**PR (95% CI)**	**PR (95% CI)**	**PR (95% CI)**	**PR (95% CI)**	**PR (95% CI)**	**PR (95% CI)**	**PR (95% CI)**	**PR (95% CI)**
**Cognitive development**									
< median	1.16 (0.98; 1.36)	0.92 (0.73; 1.16)	1.57 (0.86; 2.89)	1.06 (0.94; 1.18)	1.00 (Ref.)	0.82 (0.46; 1.47)	0.77 (0.65; 0.90)	0.98 (0.84; 1.13)	1.45 (1.06; 1.99)
≥ median	1.52 (1.06; 2.18)	1.37 (0.94; 2.00)	1.17 (0.23; 5.86)	1.21 (0.99; 1.49)	1.00 (Ref.)	1.20 (0.79; 1.81)	0.77 (0.47; 1.26)	1.13 (0.84; 1.52)	1.20 (0.67; 2.18)
p-interaction	.51	.009	.50	.22		.88	.004	<.001	.34
**Language development**									
< median	1.50 (1.30; 1.72)	1.01 (0.80; 1.28)	1.23 (0.43; 3.49)	1.26 (1.13; 1.42)	1.00 (Ref.)	0.53 (0.24; 1.18)	1.03 (0.89; 1.20)	1.03 (0.88; 1.20)	1.26 (0.78; 2.01)
≥ median	3.45 (2.28; 5.26)	2.34 (1.49; 3.67)	NS	3.03 (2.31; 4.00)	1.00 (Ref.)	1.09 (0.52; 2.30)	1.71 (0.97; 3.00)	2.04 (1.38; 3.02)	0.81 (0.21; 3.14)
$P_{interaction}$	.002	<.001	NS	<.001		.60	<.001	<.001	.38
**Socioemotional development**									
< median	1.39 (1.19;1.62)	1.13 (0.90; 1.42)	1.29 (0.45; 3.68)	1.23 (1.09; 1.39)	1.00 (Ref.)	0.87 (0.48; 1.59)	1.02 (0.87; 1.19)	0.93 (0.78; 1.10)	1.54 (1.04; 2.27)
≥ median	1.68 (1.18; 2.40)	1.10 (0.70; 1.70)	2.24 (0.78; 6.45)	1.34 (1.08; 1.67)	1.00 (Ref.)	1.34 (0.90; 2.00)	0.74 (0.42; 1.30)	1.10 (0.79; 1.54)	1.28 (0.70; 2.34)
p-interaction	.71	.67	.78	.48		.74	.30	<.001	.38
**Motor development**									
< median	1.00 (0.80; 1.24)	1.04 (0.78; 1.41)	0.73 (0.14; 3.84)	0.92 (0.78; 1.07)	1.00 (Ref.)	1.38 (0.79; 2.44)	0.99 (0.80; 1.21)	1.01 (0.83; 1.24)	2.20 (1.56; 3.11)
≥ median	0.65 (0.48; 0.88)	1.14 (0.88; 1.50)	0.71 (0.26; 1.94)	0.79 (0.68; 0.92)	1.00 (Ref.)	0.95 (0.75; 1.20)	1.14 (0.74; 1.76)	0.99 (0.76; 1.28)	0.73 (0.47; 1.13)
$P_{interaction}$	.08	.90	>.99	.35		.46	.87	.54	<.001

Data presented as prevalence ratio (PR) and 95% CIs for poor development (score <25th centile). Analyses were adjusted by age, sex, urbanicity and country of origin. Total participants: 6 815. Nutritional status was classified using weight-for-height (z-score) following the WHO recommendations. Fat was classified using triceps following the age- and sex-specific cut-off points of Frisancho 1981 (Normal: P10-P90; low <P10; High >P90).

NS: Not sufficient poor developed children for moderator analysis. SD: standard deviations.

© 2022 Petermann-Rocha F et al. *JAMA Network Open.*

eTable 8. Combined Associations of WHZ and Fat With Early Childhood Development by Country of Origin

	Wasted (WHZ < -2 SD)			Normal weight (-2 ≤ WHZ ≤ 2 SD)			Overweight (WHZ > 2 SD)		
	Low body fat	Normal body fat	High body fat	Low body fat	Normal body fat	High body fat	Low body fat	Normal body fat	High body fat
	PR (95% CI)	PR (95% CI)	PR (95% CI)	PR (95% CI)	PR (95% CI)	PR (95% CI)	PR (95% CI)	PR (95% CI)	PR (95% CI)
**Cognitive development**									
East Asia	1.30 (0.59; 2.88)	1.57 (1.00; 2.45)	0.62 (0.09; 4.18)	0.70 (0.49; 1.00)	1.00 (Ref.)	0.79 (0.51; 1.21)	NS	1.50 (0.94; 2.38)	0.62 (0.29; 1.34)
South-east Asia[a]	1.41 (0.73; 2.70)	0.45 (0.06; 3.22)	NS	1.47 (0.77; 2.79)	1.00 (Ref.)	NS	1.69 (0.74; 3.86)	NS	NS
Pacific Region[b]	1.15 (0.95; 1.40)	0.97 (0.79; 1.19)	1.34 (0.83; 2.18)	0.94 (0.84; 2.18)	1.00 (Ref.)	0.99 (0.84; 1.06)	0.78 (0.67; 0.92)	0.98 (0.86; 1.11)	1.45 (1.08; 1.94)
$P_{interaction}$[a]	.52	.32	NS	.02		NS	NS	NS	NS
$P_{interaction}$[b]	.60	.16	.39	.48		.50	NS	.06	.04
**Language development**									
East Asia	2.35 (0.58; 9.52)	2.38 (1.16; .90)	NS	0.36 (0.13; 1.03)	1.00 (Ref.)	0.94 (0.43; 2.03)	NS	1.60 (0.65; 3.91)	0.36 (0.05; 2.63)
South-east Asia[a]	0.91 (0.60; 1.39	0.48 (0.12; 1.90)	NS	0.92 (0.61; 1.39)	1.00 (Ref.)	NS	1.12 (0.66; 1.89)	0.93 (0.13; 6.59)	NS
Pacific Region[b]	1.14 (0.95; 1.38)	1.01 (0.82; 1.23)	0.89 (0.33; 2.41)	1.02 (0.91; 1.14)	1.00 (Ref.)	0.84 (0.44; 1.60)	0.89 (0.77; 1.03)	0.92 (0.80; 1.05)	1.23 (0.81; 1.89)
$P_{interaction}$[a]	.60	.06	NS	.03		NS	NS	.46	NS
$P_{interaction}$[b]	.36	.05	NS	.07		.95	NS	.19	.22
**Socioemotional development**									
East Asia	0.83 (0.29; 2.39)	1.20 (0.71; 2.03)	1.25 (0.32; 4.81)	0.46 (0.29; 0.74)	1.00 (Ref.)	0.90 (0.60; 1.35)	1.33 (0.19; 9.36)	1.38 (0.83; 2.29)	0.65 (0.30; 1.42)
South-east Asia[a]	1.91 (0.92; 3.98)	0.58 (0.08; 4.18)	NS	1.90 (0.92; 3.95)	1.00 (Ref.)	NS	2.56 (1.16; 5.66)	NS	NS
Pacific Region[b]	1.30 (1.08; 1.57)	1.11 (0.90; 1.38)	1.03 (038; 2.80)	1.09 (0.97; 1.24)	1.00 (Ref.)	1.15 (0.65; 2.03)	0.95 (0.81; 1.11)	0.91 (0.78; 1.06)	1.64 (1.20; 2.25)
$P_{interaction}$[a]	.11	.61	NS	<.001		NS	.35	NS	NS
$P_{interaction}$[b]	.54	.85	.88	.003		0.42	.60	.100	.024
**Motor development**									
East Asia	1.08 (0.66; 1.78)	1.10 (0.82; 1.46)	0.71 (0.26; 1.93)	0.98 (0.82; 1.18)	1.00 (Ref.)	0.94 (0.75; 1.19)	1.03 (0.30; 3.54)	0.80 (0.55; 1.16)	0.74 (0.48; 1.13)
South-east Asia[a]	0.81 (0.50; 1.33)	0.54 (0.14; 2.08)	NS	0.60 (0.37; 0.96)	1.00 (Ref.)	NS	0.63 (0.23; 1.72)	NS	NS
Pacific Region[b]	0.91 (0.65; 1.27)	1.19 (0.91; 1.57)	0.71 (0.13; 3.73)	1.06 (0.90; 1.25)	1.00 (Ref.)	1.30 (0.66; 2.59)	1.15 (0.94; 1.40)	1.13 (0.93; 1.36)	1.95 (1.40; 2.72)
$P_{interaction}$[a]	.76	.41	NS	.12		NS	.64	NS	NS
$P_{interaction}$[b]	.45	.54	.97	.84		.38	.99	.154	<.001

Data presented as prevalence ratio (PR) and 95% CIs for poor development (score <25th centile). Analyses were adjusted by age, sex, SES and urbanicity. Total participants: 6 815.
Nutritional status was classified using weight-for-height (z-score) following the WHO recommendations. Fat was classified using triceps following the age- and sex-specific cut-off points of Frisancho 1981 (Normal: P10-P90; low <P10; High >P90). [a] interactions between East Asia and South-east Asia; [b] interactions between East Asia and the Pacific Region.
East Asia: China and Mongolia; Southeast Asia: Cambodia, PNG, and Vanuatu
NS: Not sufficient poor developed children for moderator analysis. SD: standard deviations.

© 2022 Petermann-Rocha F et al. *JAMA Network Open.*
